# Stable expression of silencing‐suppressor protein enhances the performance and longevity of an engineered metabolic pathway

**DOI:** 10.1111/pbi.12506

**Published:** 2015-12-02

**Authors:** Fatima Naim, Pushkar Shrestha, Surinder P. Singh, Peter M. Waterhouse, Craig C. Wood

**Affiliations:** ^1^ CSIRO Agriculture Canberra ACT Australia; ^2^ School of Biological Sciences The University of Sydney Sydney NSW Australia; ^3^ School of Molecular Bioscience The University of Sydney Sydney NSW Australia; ^4^ Present address: The Centre for Tropical Crops and Biocommodities Queensland University of Technology Brisbane Qld Australia

**Keywords:** metabolic engineering, viral silencing‐suppressor proteins, long chain polyunsaturated fatty acid, transgene longevity

## Abstract

Transgenic engineering of plants is important in both basic and applied research. However, the expression of a transgene can dwindle over time as the plant's small (s)RNA‐guided silencing pathways shut it down. The silencing pathways have evolved as antiviral defence mechanisms, and viruses have co‐evolved viral silencing‐suppressor proteins (VSPs) to block them. Therefore, VSPs have been routinely used alongside desired transgene constructs to enhance their expression in transient assays. However, constitutive, stable expression of a VSP in a plant usually causes pronounced developmental abnormalities, as their actions interfere with endogenous microRNA‐regulated processes, and has largely precluded the use of VSPs as an aid to stable transgene expression. In an attempt to avoid the deleterious effects but obtain the enhancing effect, a number of different VSPs were expressed exclusively in the seeds of *Arabidopsis thaliana* alongside a three‐step transgenic pathway for the synthesis of arachidonic acid (AA), an ω‐6 long chain polyunsaturated fatty acid. Results from independent transgenic events, maintained for four generations, showed that the VSP‐AA‐transformed plants were developmentally normal, apart from minor phenotypes at the cotyledon stage, and could produce 40% more AA than plants transformed with the AA transgene cassette alone. Intriguingly, a geminivirus VSP, V2, was constitutively expressed without causing developmental defects, as it acts on the siRNA amplification step that is not part of the miRNA pathway, and gave strong transgene enhancement. These results demonstrate that VSP expression can be used to protect and enhance stable transgene performance and has significant biotechnological application.

## Introduction

The introduction of complex transgenic pathways specifically expressed in oilseeds to generate modified oils has long been a target of plant genetic engineers, producing a range of nutritional, pharmaceutical and industrial oils (Cahoon and Kinney, 2005; Nykiforuk *et al*., 2012; Petrie *et al*., 2012; Vanhercke *et al*., 2013). The process of generating elite transgenic events in many crops is arduous (Saunders and Lomonossoff, 2013) and from the large population of transgenic events produced in the initial tissue culture stage, only a small subpopulation give high levels of transgene performance that are stable in subsequent generations (Hagan *et al*., 2003). It has been suggested that high transgene expression triggers silencing pathways and that initial highly expressing events soon become incapacitated (Lindbo *et al*., 1993; Saunders and Lomonossoff, 2013; Schubert *et al*., 2004). It is clear that this silencing operates at both the transcriptional and post‐transcriptional level and is guided by small RNAs (sRNAs) (Hagan *et al*., 2003; Schubert *et al*., 2004).

Plant viruses have evolved ways to evade or negate the host's silencing mechanisms. They deploy a range of strategies, including the expression of viral silencing‐suppressor proteins (VSPs). These proteins interfere with silencing pathway components, sometimes targeting a number of them simultaneously (Ding and Voinnet, 2007; Incarbone and Dunoyer, 2013). They therefore have great potential as enhancers of transgene expression. However, normal plant growth and responses to the environment are also dependent on sRNA‐guided processes, so the generic inhibition or ablation of sRNAs to prevent gene silencing can have developmental and defence‐response consequences. We sought to find a VSP, or a way of applying it, that enhanced stable transgene expression without deleterious side effects. In our study, we examined three VSPs: p19, V2 and P0^PE^.

Tombusvirus p19 is a small protein encoded by *Tomato bushy stunt virus* (TBSV) (a *positive‐strand RNA virus*). It was one of the first VSPs used in transient leaf assay systems to inhibit the post‐transcriptional gene silencing of transgenes (Voinnet *et al*., 2003). This small (19 kDa) and soluble protein has been extensively studied, including the resolution of crystal structures of p19 bound to sRNA. In conjunction with *in vitro* and *in vivo* experiments (Silhavy *et al*., 2002; Ye *et al*., 2003), the crystal structures show that p19 forms a homodimer specifically with 21 nt sRNA duplexes that contain 2 nt 3′ overhangs (Lakatos *et al*., 2004; Vargason *et al*., 2003; Ye *et al*., 2003). Constitutive expression of p19 with *Cauliflower mosaic virus* (CaMV) 35S promoter causes major developmental defects in *A. thaliana*, including flower abnormalities and leaf serration (Dunoyer *et al*., 2004). The V2 VSP is encoded by the *Tomato yellow leaf curl virus* (TYLCV), a single‐stranded DNA begomovirus. In the Israeli isolate of TYLCV, V2 is well characterized and mutation of it results in loss of viral infection in *Nicotiana benthamiana* and tomato (Wartig *et al*., 1997). V2 is involved in systemic movement of the virus (Wartig *et al*., 1997) and also allows overexpression of GFP in transient assays (Zrachya *et al*., 2007). The direct interaction between V2 and SGS3 has been proposed to disrupt the RDR6‐driven production of sRNA against TYLCV (Glick *et al*., 2008). However, Fukunaga and Doudna (2009) reported that V2 shares RNA binding selectivity with SGS3 and that it outcompetes SGS3 in binding double‐stranded RNA structures that have 5′ overhangs. Lastly, P0 proteins are encoded by poleroviruses and enamoviruses, which are in the *Luteoviridae* family of plant viruses. P0^PE^ is encoded by the first open reading frame of enamovirus *Pea enation mosaic virus‐1* (PEMV‐1). Fusaro *et al*. (2012) reported that P0^PE^ acts as an F‐Box protein and that its constitutive expression in *A. thaliana* causes major developmental defects. Polerovirus P0 and enamovirus P0^PE^ proteins have been shown to inhibit production of secondary sRNA through destabilization of AGO1 (Bortolamiol *et al*., 2007; Fusaro *et al*., 2012). These three VSPs are encoded by phylogenetically unrelated viruses and have different modes of action, yet they all display the common feature of enhancing transgene expression in transient assay expression systems (Fusaro *et al*., 2012; Incarbone and Dunoyer, 2013; Voinnet *et al*., 2003; Zrachya *et al*., 2007).

The trait that we sought to enhance by judicious use of a VSP was the production of arachidonic acid (AA), an ω‐6 long chain polyunsaturated fatty acid (LCPUFA) required for brain development in infants and the precursor for a number of mammalian signalling compounds (Spychalla *et al*., 1997). It is made of a 20‐carbon chain with four double bonds and synthesized in some micro‐organisms from linoleic acid by a 3‐step pathway involving an elongase and two desaturases. Plants do not contain AA; however, introducing transgenes for these enzymes into *A. thaliana* has produced lines that accumulate up to 20% AA in their seed oil profiles (Petrie *et al*., 2012). The promoters used in driving transgene expression in seeds are chosen from pathways that are exclusively active in seed protein and oil storage, a period of development distinct from early embryogenesis (Belmonte *et al*., 2013; Le *et al*., 2010). Here, we investigate whether VSP expression can be tolerated and can enhance transgene activity in plants during late seed development.

## Results

### Effects of expressing VSP on development and seed oil composition in *Arabidopsis thaliana*

**Figure 1:**
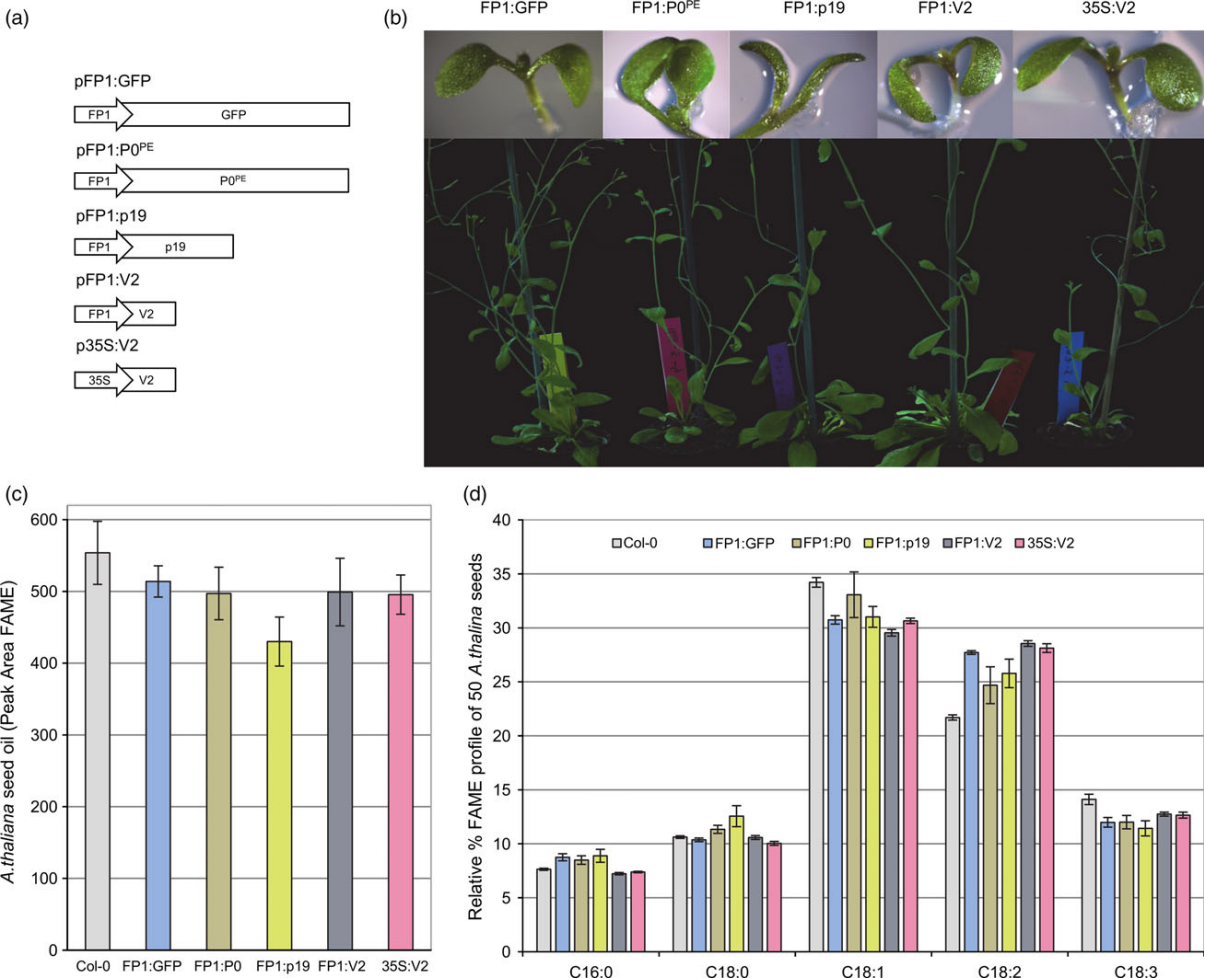
Phenotypic and oil profile of transgenic *Arabidopsis thaliana* lines expressing various VSPs. (a) Schematic of T‐DNA binary plasmids designed for stable expression of the VSPs (P0 ^PE^ p19 and V2) in *A. thaliana*. Genes driven by the *Brassica napus* truncated napin FP1 promoter and the CaMV 35S promoter as indicated. For comparison purposes, a control construct encoding GFP driven by the FP1 promoter was also designed. (b) The phenotypic changes in cotyledons of *A. thaliana* stably transformed with constructs outlined in (a). Representative mature plants expressing the respective constructs outlined in (a). Images presented are of stably transformed *A. thaliana* in the third generation.(c–d) Total oil and relative percentages of the major fatty acids extracted as fatty acid methyl esters (FAME) from seeds of Col‐0 and T3 transgenic *A. thaliana*. Data presented are from 3 independent events for each plasmid described in Figure 1a. Error bars are the standard error of the mean.

To test whether seed‐specific expression of a VSP gives localized enhanced transgene expression without causing developmental defects, the napin FP1 promoter from *Brassica napus* was investigated. FP1 has been reported to give high level of expression throughout the oil storage and protein synthesis stages of seed development but not during critical events of embryogenesis (Le *et al*., 2010). First, a construct with GFP under the control of the FP1 promoter was made (Figure 1) and transformed into *A. thaliana*. Then the promoter was placed upstream of the coding region of one of three different VSPs (V2, p19 and P0^PE^; Figure 1a) and these constructs transformed into *A. thaliana*. A constitutive promoter, CaMV 35S, was also used to drive V2. Visual observation under blue light, and Western blot analysis (Figure S1), of T2 FP1:GFP lines confirmed that the GFP expression was restricted to the seed; it was not observed in roots, cotyledons, true leaves or shoot apical meristems. The pFP1:P0^PE^ construct gave relatively few transgenic lines compared to the numbers obtained using pFP1:V2 and pFP1:p19 constructs; the FP1:P0^PE^ and FP1:p19 lines displayed altered cotyledon phenotypes (Figure 1b). Despite this, the transgenic lines from all three constructs grew vigorously and were fertile.

The composition and levels of lipids in 50‐seed samples from three independent T3 lines per genotype were quantified (Figure 1c & 1d). FP1:P0^PE^ and FP1:V2 lines had slightly reduced total lipid levels compared to wild‐type Col‐0, but these were no more reduced than in FP1:GFP. However, FP1:p19 seeds had lower total lipid levels than those in FP1:GFP plants, suggesting that p19 expression has an adverse effect on seed oil biosynthesis (Figure 1c). Examining the effects on the canonical *A. thaliana* seed oil pathway revealed that the oleic acid (18:1) levels in FP1:GFP, FP1:p19 and FP1:V2 were decreased by ~4%, whereas the linoleic acid (18:2) levels in FP1:GFP and FP1:V2 increased by ~6% and in FP1:P0^PE^ and FP1:p19 by ~4%. The relative percentage of linolenic acid (18:3) decreased by ~2% in all lines (Figure 1d).

Overall, the expression of the VSPs, in the absence of a transgenic oil modification pathway, caused little or no developmental defects and only minor changes in seed oil composition and content.

### An intergenerational study of populations of transgenic *Arabidopsis thaliana* expressing AA and VSP in seed

**Figure 2:**
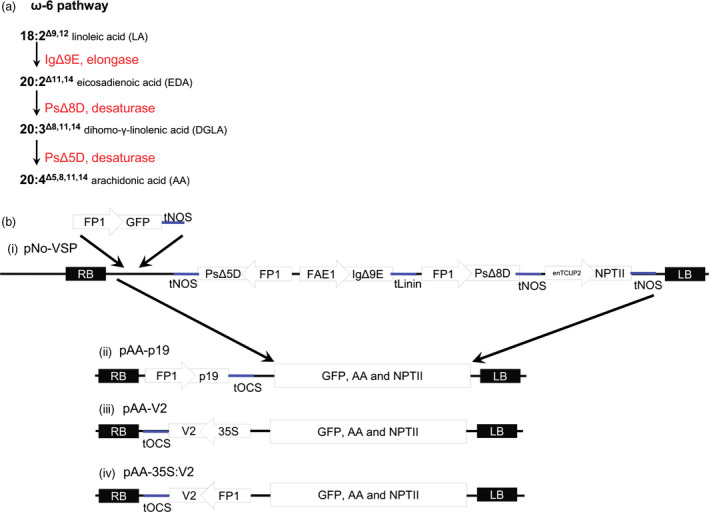
Design of constructs containing a three‐gene pathway for biosynthesis of AA (20:4) in seed oil. (a) A schematic of enzymes involved in the biosynthesis of 20:4 via the ω‐6 pathway using 18:2 (LA) as the starting substrate. Abbreviations are as follows: IgΔ9E, *Isochrysis galbana* Δ9‐elongase; PsΔ8D, *Pavlova salina* Δ8‐desaturase; PsΔ5D, *Pavlova salina* Δ5‐desaturase. (b) A schematic of the construct maps designed for stable transformation of *Arabidopsis thaliana*. (i) The construct pNo‐VSP consisted of the *IgΔ9E* driven by the *A. thaliana* FAE1 promoter, *PsΔ8D* and *PsΔ5D* and *GFP* driven by the FP1 promoter. (ii–iv) Addition of a VSP to pNo‐VSP generated pAA‐p19, pAA‐V2 and pAA‐35S:V2. Abbreviations are as follows: RB, right border; tOCS,* Agrobacterium tumefaciens* octopine synthase terminator; 35S, *Cauliflower mosaic virus* 35S promoter; GFP, green fluorescent protein reporter; tNOS,* A. tumefaciens* nopaline synthase terminator (polyadenylation signal); tLinin, *Linum usitatissimum* conlinin2 terminator; enTCUP2, enhanced tobacco cryptic promoter 2; NPTII, neomycin phosphotransferase II selectable marker; LB, left border.

To provide a three‐transgene metabolic pathway, with which to evaluate the effects of VSP expression, the single AA pathway cassette of Petrie *et al*. (2012) was utilized. This contains the fatty acid biosynthesis genes *Isochrysis galbana* Δ9‐elongase (*IgΔ9E*), *Pavlova salina* Δ8‐desaturase (*PsΔ8D*) and *Pavlova salina* Δ5‐desaturase (*PsΔ5D*) (Figure 2a). *IgΔ9E* is driven by the *A. thaliana* FAE1 promoter and *PsΔ8D* and *PsΔ5D* are both driven by the FP1 promoter (Figure 2b). The FP1:GFP:Nos was inserted to the original triple gene construct, then the cassette containing these and the NPTII gene was excised and inserted into pFP1:p19 and pFP1:V2 to generate pAA‐p19 and pAA‐V2, (Figure 2b). P0^PE^ was excluded in this part of the study due to the low number of transformants obtained earlier. The GFP:AA intermediate construct was retained, termed ‘pNo‐VSP’ and subsequently used as control. All of these T‐DNA binary constructs were transformed into *A. thaliana* genotype MC49, which is a *fad3/fae1* double mutant containing high levels of 18:2 in seed oil (Zhou *et al*., 2006). The 18:2 fatty acid is the starting substrate for the introduced transgenic pathway.

Fifteen independent T1 lines (Table 1) were randomly selected, for each AA construct, from a large number of primary transformants, and followed for four generations. Segregation analysis was performed for T2 (Figure S2) and T3 generations. This analysis showed that the segregation pattern was relatively similar in all transgenic populations, that the vast majority of the VSP expressing lines are not multiloci insertion events (Figure S2), and that by T3 11/15 No‐VSP, 13/15 AA‐p19, and 11/15 AA‐V2 lines were homozygous (Figure S3).

**Table 1:** Summary of the transgenic *Arabidopsis thaliana* population generated with the AA constructs

Plasmid	Independent T1 events	Events taken to T5
pNo‐VSP	24	15
pAA‐p19	39	15
pAA‐V2	26	15
pAA‐35S:V2	8	5

**Figure 3:**
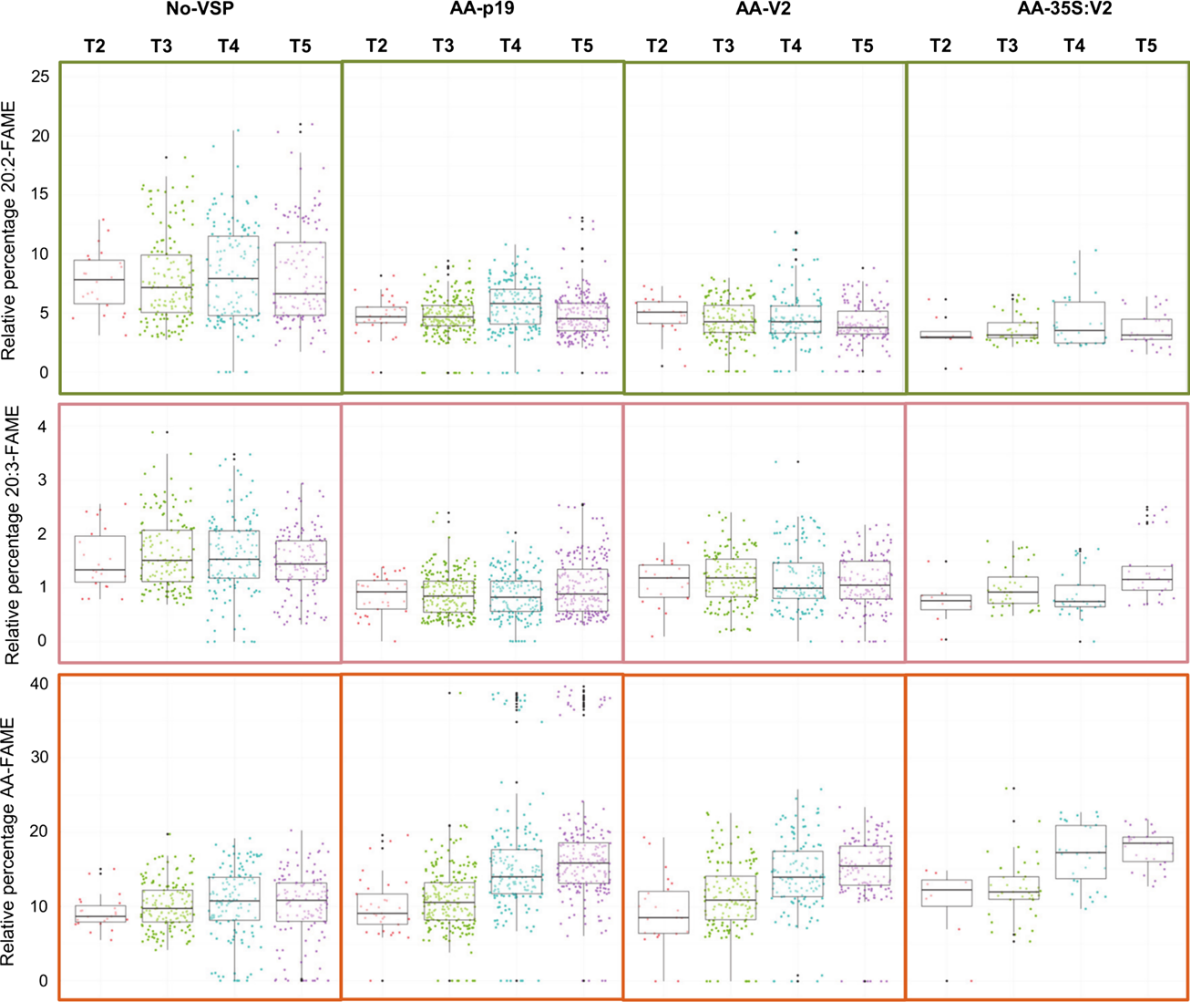
Relative percentages of 20:2‐FAME, 20:3‐FAME and AA‐FAME extracted from T2–T5 seeds of transgenic *A. thaliana*. Box‐whisker plot analysis of a large population of transgenic *A. thaliana* expressing the constructs described in Figure 2. Relative percentages of 20:2, 20:3 and AA shown and dot points refer to independent transformation events in T2 and their respective progenies in T3–T5. Points outside of the whiskers are not included in the calculation of median or quartiles.

The table summarizes the generation of independent transgenic events for each plasmid. *Arabidopsis thaliana* was transformed with these plasmids and a large number of independent events were generated. Each data point for parent and descendent was preserved and graphed, generating box‐whisker plots in Figure 3 and progeny plots in Figure S3.

**Figure 4:**
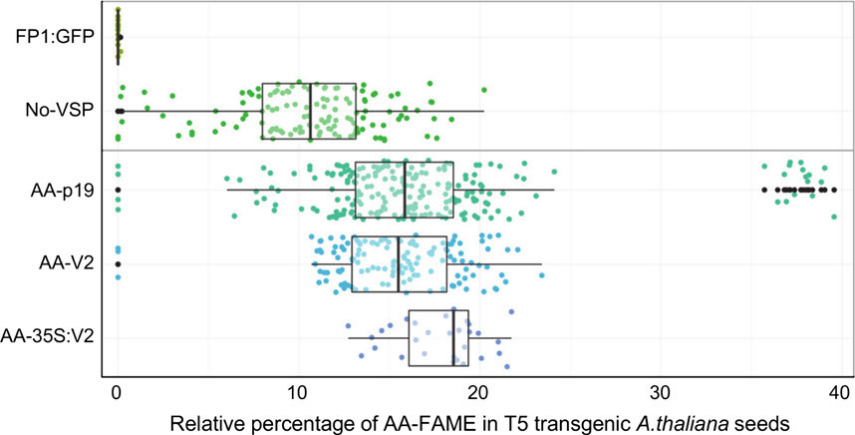
Relative percentages of AA‐FAME extracted from T5 seeds of transgenic *Arabidopsis thaliana*. Box‐whisker plot analysis of a T5 population of transgenic *A. thaliana* expressing the constructs described in Figure 2. Relative percentage of AA measured in T5 seeds and each line represented as a dot point. FP1:GFP is the negative control of MC49 transformed with pFP1:GFP plasmid.

In each generation, the metabolic profiles of seed samples were measured for 15 individual progeny (Figures 3 and 4). The expression of either V2 or p19 improved the population median for AA in T5 and one elite event, AA‐p19‐26 (Figure S3), produced 39–41% AA in T3, T4 and T5 seeds. The V2 and p19 lines with high levels of AA, had low levels of the pathway intermediates, 20:2 and 20:3 (Figure 3). This is consistent with efficient metabolic flux of 18:2 through to AA and indicates that all three transgene‐encoded enzymes were working efficiently. The poorly performing lines generally accumulated higher levels of 20:2, indicating that transgenic constraints were occurring at the first desaturation step, IgΔ8D. For all VSP‐transgenic populations the overall trend was for an increase in the median level of AA production over generations 2, 3 and 4. The No‐VSP lines stabilized for AA production in the T4 and T5 generations, whereas AA‐p19 or AA‐V2 events continued to improve in overall production of AA in the T5 generation (Figure 3).

Segregation analysis shows that by T3 most of the lines are homozygous for the transgene cassette and strongly suggests that the jump of AA level between T2 and T3 is due to the lines transitioning from the hemizygous to homozygous state. From T3 onwards the AA levels of most No‐VSP lines decline, indicative of transgene silencing whereas most of the AA‐p19 and AA‐V2 lines maintain their elevated levels.

All data for each transgenic population at the T5 seed stage is shown in Figure 4. The T5 transgenic populations of AA‐V2 and AA‐p19 show a median of 15% and 16% AA, respectively, whereas the No‐VSP population shows a median of ~11% AA. The data for the populations also revealed that the first and third quartiles were more compact for the AA‐V2 or AA‐p19 events, compared to a wider spread in No‐VSP events.

### The effect of high levels of AA on total seed oil and seed germination and seedling vigour

**Figure 5:**
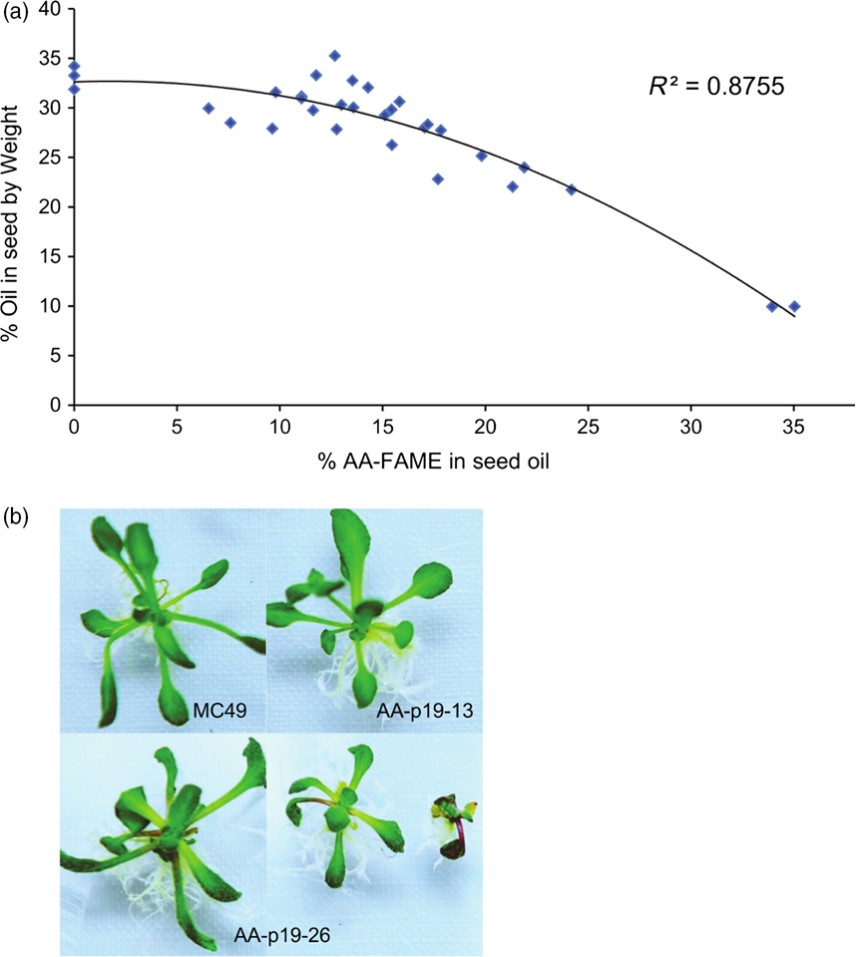
The effect of high levels of AA on the accumulation of oil (TAG) in the seed and seedling vigour. (a) Total oil extracted from T4 transgenic *A. thaliana* lines expressing pAA‐p19. Independent lines analysed were selected based on the varied levels of AA to determine a relationship between seed oil and relative percentage of AA. Oil also extracted from seeds of wild‐type MC49 and MC49 expressing FP1:GFP as controls. (b) The phenotypic comparison between AA‐p19‐26, AA‐p19‐13 and MC49 seedlings. The relative sizes and leaf shapes of AA‐p19‐26 seedlings (~38% AA) compared to AA‐p19‐13 (11% AA) and MC49 seedlings. Seedlings photographed 3 weeks after imbibition.

Various phenotypic characteristics were measured in seeds containing varying amounts of AA. In early generations, it was observed that seeds with high levels of AA were smaller than wild‐type MC49 seeds. This was investigated further by quantifying the seed oil content of independent events with levels of AA ranging from 0 to 38% as a percentage of total fatty acids (Figure 5). The nontransgenic MC49, FP1:GFP expressing MC49 and AA‐V2‐03 (null segregant) seeds contained 33%, 32% and 34% oil, respectively, indicating that the transgenesis process had not affected the seed oil profiles. Seeds containing up to ~15% AA had negligible reductions in seed oil content, however at higher levels of AA, the oil content was reduced (Figure 5a). This trade‐off between AA and oil content was most pronounced in a line (AA‐p19‐26) containing ~38% AA that resulted in a 70% reduction in oil content (Figure 5a).

Slower germination, reduced seedling vigour and small seed phenotypes were observed in a number of AA lines, independent of the presence or absence of a VSP but correlated with LCPUFA content. The seedlings of wild‐type MC49 (0% AA) and AA‐p19‐13 (11% AA) developed normally, however AA‐p19‐26 (38%) grew more slowly (Figure 5b) and produced seed with 50% viability. This observation was recorded in all later generations as well (data not presented).

### The effect of constitutive expression of V2 on plant development and AA pathway

Transgenic plants constitutively expressing VSPs, including p19, and P0, show severe development abnormalities (Dunoyer *et al*., 2004; Fusaro *et al*., 2012) but there have been no reports of plants with constitutive transgenic expression of V2. We have shown previously that V2 expression has the rare attribute of simultaneously allowing hairpin RNA‐directed silencing and enhancing overexpression of transgenes in transient Agro‐infiltration assays of *Nicotiana benthamiana* leaves (Naim *et al*., 2012). Therefore, we investigated the effect of constitutive expression of V2 on plant development and on the transgenic oil modification pathway. Wild‐type Col‐0 and MC49 *A. thaliana* were transformed with p35S:V2 and pAA‐35S:V2, respectively (Figures 1b and 2b) and transgenic populations followed for four generations. The 35S:V2 lines had no obvious phenotypic abnormality or changes in oil lipid profile (Figure 1b–d) and the AA‐35S:V2 population produced AA at ~19% in the T5 seed (Figures 3 and 4). Although fewer lines were obtained with pAA‐35S:V2 (Table 1).

## Discussion

The ubiquitous expression of VSPs (e.g. driven by the CaMV 35S promoter) usually leads to abnormal plant development and embryo lethality (Dunoyer *et al*., 2004; Fusaro *et al*., 2012) due to their effects on endogenous miRNA‐regulated functions. To avoid this, but capture VSP enhancement of transgene expression, a previous study has developed a mutated form of p19 that retains some silencing‐suppressor activity yet does not affect plant development (Saxena *et al*., 2011). Our alternative approach has been to investigate the use of fully functional VSPs expressed only in tissues that are important for seed oil production but not development. Using the FP1 promoter to drive p19 expression greatly enhanced the efficacy and longevity of the oil biosynthesis transgene cassette, as measured by the production of AA. Its seed‐specific expression pattern almost completely avoided the developmental problems encountered when constitutively expressed (Dunoyer *et al*., 2004). We conclude that the FP1 promoter is active in the cotyledon tissue, causing both the modified seed oil enhancement and the unusual cotyledon phenotype of seedlings, but not in the meristem, thus avoiding disruption of sRNA driven regulatory pathways in the shoot apex.

The seed‐specific expression of V2 or p19 not only improved the transgenic biosynthesis of AA but also maintained this effect for four generations. A possible mechanism for the initial VSP enhancement might have been by favouring the selection of multiple transgene insertion lines (which may otherwise have been rapidly silenced), especially as *A. thaliana* transformation is by floral dip and selection at the germinating seed stage. However, the range in transgene locus number, as judged from segregation ratios, was very similar for VSP and no‐VSP lines (Figure S2). This suggests that the VSP is having an enhancing effect through prevention of silencing of predominantly single loci rather than by increasing the number of transgene loci per plant.

The continued increase in AA content from T2 to T5 in the populations of VSP lines, but lack of increase in the no‐VSP lines, seems to result from the plants’ responses to transgene homozygosity. In the No‐VSP lines, the transgenes becoming homozygous trigger their own silencing, resulting in a reduction of AA biosynthesis, whereas the gains in transgene expression from homozygosity are protected in VSP plants. As the population of a VSP line becomes 100% homozygous, the cumulative effect of doubling transgene loci is reflected in increasing AA content.

Interestingly, the best performing lines had low levels of intermediates whereas the poor performing lines showed accumulation of 20:2. This suggests that the PsΔ8D transgene is most sensitive to silencing or is the bottleneck step of the pathway. While fatty acid elongation reactions are generally considered to occur in the CoA pool (Fehling and Mukherjee, 1991), desaturation reactions, such as PsΔ8D and PsΔ5D, are typically considered to operate on the phosphatidylcholine (PC) pool. In metabolic pathways that are sequential, such as for the conversion of 18:2 to AA, there is an implied movement of acyl chains between different lipid head groups in subcellular compartments. In *A. thaliana* the LCPUFA intermediates may be preferentially channelled towards AA. At the upper most extreme of metabolic capacity recorded in this study, one event was capable of ~40% AA, a level considerably higher than previously reported (Petrie *et al*., 2012). However, high levels of LCPUFA in *A. thaliana* resulted in reduced oil content and reduced seedling vigour. The production of exotic fatty acids in seeds often results in compromised seed oil content (van Erp *et al*., 2011; Nykiforuk *et al*., 2012) most likely due to the inefficient flux of new fatty acids from their site of synthesis into storage oils and altered properties of the membranes in the developing seed (Thomæus *et al*., 2001). Previous reports show that the production of a novel fatty acid, ricinoleic acid, in *A. thaliana* resulted in poor oil content (van Erp *et al*., 2011). It has been demonstrated that the reduced oil content could be recovered with the inclusion of more specialized oil accumulation enzymes specific for transfer of ricinoleic acid into seed oil (van Erp *et al*., 2015). Similar strategies could be employed to recover the total oil content in plants generated in this study that produce very high levels of AA.

The seed‐specific expression of V2 or p19 could easily be combined with transgenic pathways to produce high levels of other biocommodities, especially those that do not have an adverse effect on seed germination. Bio‐products such as recombinant antibodies and vaccines have been produced in leaf, tuber, and seeds (Bortesi *et al*., 2009; Magnusdottir *et al*., 2013; Schünmann *et al*., 2002) at sufficient levels for commercial production. Using our approach of expressing the bio‐product transgene and a VSP in seeds may significantly further enhance the bio‐product yield. Moreover, because V2 can be expressed without side effects in the whole plant this has the potential to enhance the other nonseed transgenic expression systems. A great concern when taking a transgenic trait into the field for commercial production is that the trait will be lost over subsequent generations. It takes at least 7–10 generations from production of the primary transformant to the first generation for commercial sale. Our results suggest that inclusion of VSP expression is preventing a decline in gene expression over generations. Such surety of performance is likely to be highly prized in agribusiness.

## Experimental procedures

### Molecular cloning – Preparation of constructs

Each VSP was cloned under the expression control of the FP1 (Stalberg *et al*., 1993) and *OCS*3′ transcription termination/polyadenylation region with the exception of 35S:V2 where the V2 coding region was under the control of the CaMV 35S promoter. The fatty acid biosynthesis genes *IgΔ9E*,* PsΔ8D* and *PsΔ5D* and the bacterial selection marker were obtained on a single DNA fragment from pJP3010 (Petrie *et al*., 2012) by digestion with *Pme*I and *Avr*II giving rise to a 9560 bp fragment. The three fatty acid biosynthesis genes on this fragment were oriented and spaced in the same manner as in pJP107 (Petrie *et al*., 2012). Binary plasmids of pFP1:p19, pFP1:V2 and p35S:V2 were digested with SwaI and AvrII. The 9560 bp of pJP3010 was ligated to the ~8000 bp fragment of the VSP containing plasmids (T4 DNA Ligase – Promega, Sydney, NSW, Australia) and electroporated into DH5α. This gave rise to three plasmids namely pAA‐p19, pAA‐35:V2 and pAA‐V2. The pAA‐p19 plasmid was digested with AhdI to drop out the FP1:p19 cassette. The backbone was re‐cirularized to form the pNo‐VSP plasmid. Plasmids comprised an *NPT*II selectable marker gene within the T‐DNA and adjacent to the left border and an *RK2* origin of replication for maintenance of the plasmids in *Agrobacterium tumefaciens*. The newly prepared vectors were electroporated into GV3101 strain of *A. tumefaciens* for stable transformation of *A. thaliana*.

### Transformation of *Arabidopsis thaliana* with the VSP and AA constructs

To transform *A. thaliana*, plants were treated by the floral dip method (Clough and Bent, 1998). The treated plants were grown to maturity and T1 seeds harvested from them were plated on MS media (Murashige and Skoog, 1962) containing kanamycin. Screening for GFP expression in the seed was also used as a visual marker for selection of T1 seeds. The seedlings that survived on MS media containing 50 mg/L of kanamycin or which were obtained from GFP‐positive seeds were transferred to soil and grown to maturity for T2 seeds.

### Segregation analysis and plant growth conditions

Segregation analysis was also carried out for each individual event by growing 60‐70 seeds on MS media containing kanamycin (Figure S2). Six to ten positive seedlings from each event were transferred to soil and grown to maturity. Plants were grown with an 8/16 h (light/dark) photoperiod in 22 °C. The *A. thaliana* plants generated during this study were grown in the same PC2 growth room with unchanged conditions throughout the two‐year period.

### Lipid analyses

Approximately 100 pooled seeds of transgenic *A. thaliana* were taken from each transformed plant for the determination of seed fatty acid composition. FAME (fatty acid methyl esters) was prepared with 750 μL of MeOH:HCl:DCM (10:1:1) containing 2 μg of internal standard (17:0 free fatty acid; NU‐CHECK PREP, Inc.). Samples were heated at 80 °C for 2 h. After cooling, 300 μL of MQ water and 500 μL of Hexane:DCM (4:1) were added to each sample, which was then vortexed and layers allowed to separate. In most cases, it was not necessary to centrifuge. The top organic layer was extracted into a clean vial and evaporated under N_2_ gas, and FAME was dissolved in 200 μL of hexane. Gas chromatography analysis performed as described previously (Wood *et al*., 2009). Each run included a freshly diluted 411 standard (Nu‐Check Prep Inc, Elysian, MN, USA) used for normalization of FAME profiles and fatty acid composition analysed as described previously (Wood *et al*., 2009).

For the quantification of oil, seeds were cleaned to remove debris and desiccated for 24 h. Approximately 5 mg of seeds was weighed using a precision balance (Mettler Toledo) and transferred to 2 mL glass vial. The addition of a known amount of internal standard (heptadecanoin; Nu‐Check Prep Inc, Elysian, MN, USA) and seed mass were used to calculate amount of oil/mg of seed. FAME was prepared by incubating the seed samples in 0.7 mL of 1N Methanolic‐HCl (Supelco) for 2 h at 80 °C. After incubation, the vials were cooled to room temperature followed by addition of 0.35 mL of 0.9% NaCl and 0.1 mL hexane and mixed in a shaker (Vibramax) for 10 min. The vials were centrifuged at 1700 *g* for 5 min and the upper phase containing FAME was transferred to a new vial and analysed by GC. Oil content (TAG) in the seeds was calculated as the sum of glycerol‐ and fatty acyl moieties using a relation:%TAG by weight = 100 × ((41 × total mol FAME / 3) + (total g FAME − (15 × total mol FAME))) / g seed dry weight, where 41 and 15 are molecular weights of glycerol moiety and methyl group, respectively.

FW10‐23 is the elite T3 control line generated by Petrie *et al*. (2012). Therefore, to monitor deviations in sample preparation and the sensitivity of analytical instruments, FAME was extracted from seeds of FW10‐23 and analysed in every batch of transgenic *A. thaliana* during the two‐year period. Statistical analyses were performed on the FAME profile of FW10‐23 collected during data collection period and there were no statistically significant differences.

### Western blot analysis

Total proteins were separated on a 12% SDS‐PAGE gel, transferred to Immobilon P membrane (Millipore, Bedford, MA) and detected by chemiluminescence. GFP was detected with anti‐GFP monoclonal antibody (Clontech, 1:5000, Clayton, Victoria, Australia) followed by anti‐mouse Ig HRP conjugate (Promega, 1:5000).

## Supporting information


**Figure S1** Activity of FP1 promoter measured in different tissues of *Arabidopsis thaliana*.
**Figure S2** Dot plot of the percentage of kanamycin resistant T2 seedlings expressing AA.
**Figure S3** The progeny plots for the various transgenic populations of *Arabidopsis thaliana* connecting T2 events to their respective progeny in T3, T4 and T5.
