# Supplementary material for: Stable expression of silencing‐suppressor protein enhances the performance and longevity of an engineered metabolic pathway
Source: Plant Biotechnol J. 2015 Dec 2;14(6):1418–26. doi: 10.1111/pbi.12506 (PMC5063197; doi:10.1111/pbi.12506)

# Supplementary Figures

**Supplementary Figure 1. Activity of FP1 promoter measured in different tissues of *A.thaliana*.**

Western blot analysis of GFP expression in roots, cotyledons, true leaves, meristem and seeds of transgenic *A.thaliana* expressing pFP1:GFP. The lower panel shows total protein loading (coomassie stain of duplicate gel) and the upper panel shows signal generated by an antibody recognising GFP, anti-GFP.


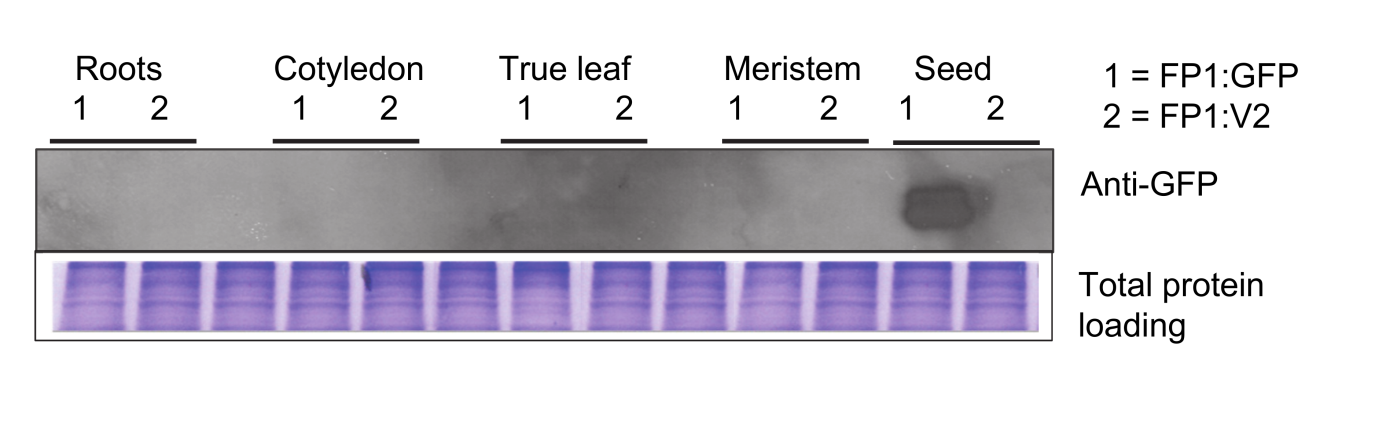


**Supplementary Figure 2. Dot plot of the percentage of kanamycin resistant T2 seedlings expressing AA.**

60–70 seeds of each T2 line were germinated on MS media containing kanamycin. The kanamycin resistant seedlings were counted and a percentage was calculated to determine segregation ratios. A single locus insertion line would be expected to give 75% Kanamycin-resistant progeny. Two or more insertions would give > 94%.

**
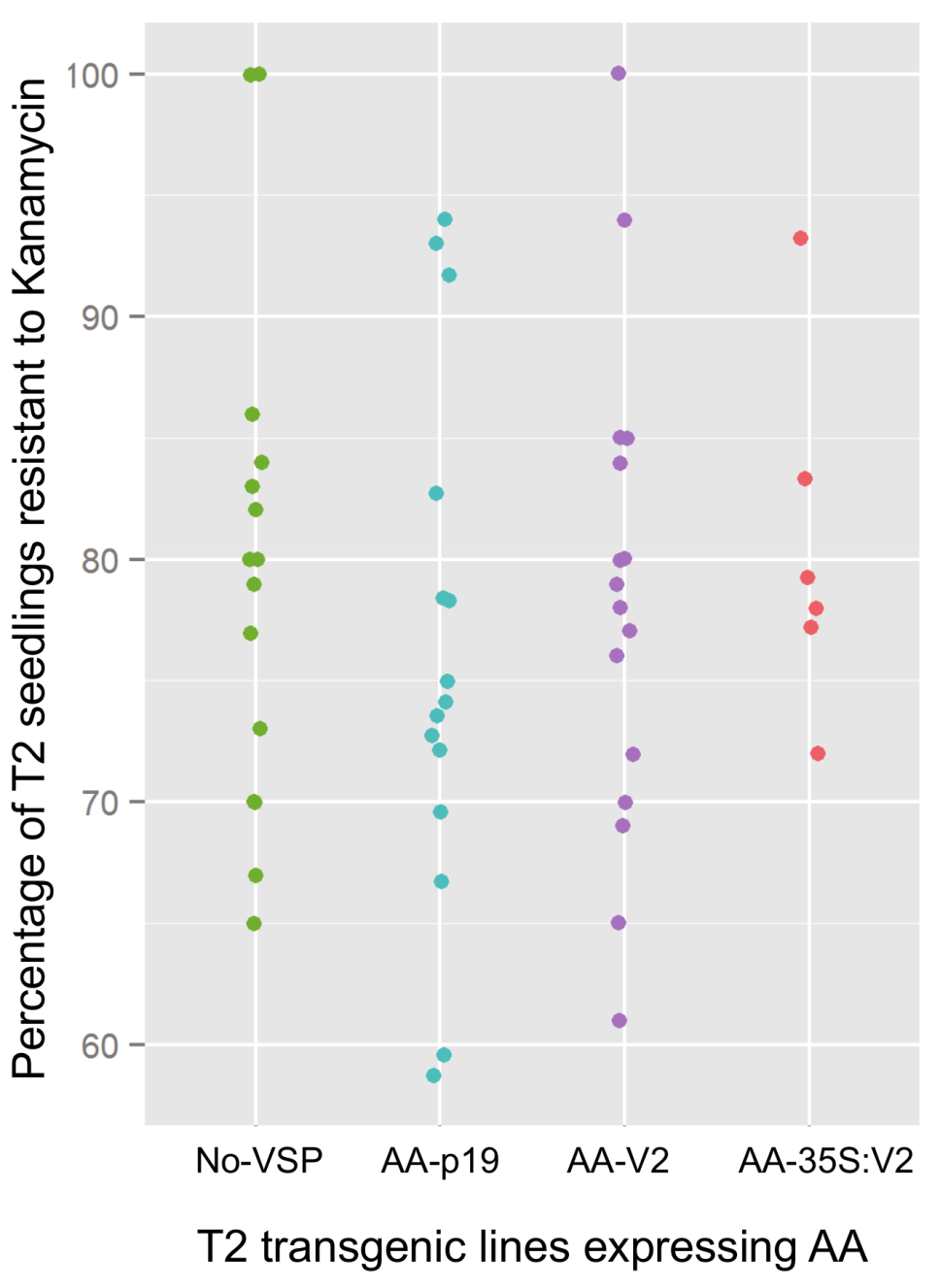
**

**Supplementary Figure 3. The progeny plots for the various transgenic populations of *A.thaliana* connecting T2 events to their respective progeny in T3, T4 and T5.**

**(a)** The progeny plots of lines transformed with plasmid pNo-VSP. “No-VSP-01” panel will be used to explain the basis of the progeny plots. No-VSP-01 shows the percentage of AA-FAME extracted from T2 – T5 seeds and each result is plotted as a dot (y-axis is the percentage of AA-FAME and x-axis is the generation of seed). The dots are then connected with lines to show the connection of parent line to progeny. Six to ten progeny lines of a parent were grown to the next generation and FAME extracted from pooled seed of each line. The lines that became homozygous in T3 are indicated by a star.

**(b)** The progeny plots of lines transformed with plasmid pAA-p19.

**(c)** The progeny plots of lines transformed with plasmid pAA-V2.

**(d)** The progeny plots of lines transformed with plasmid pAA-35S:V2.


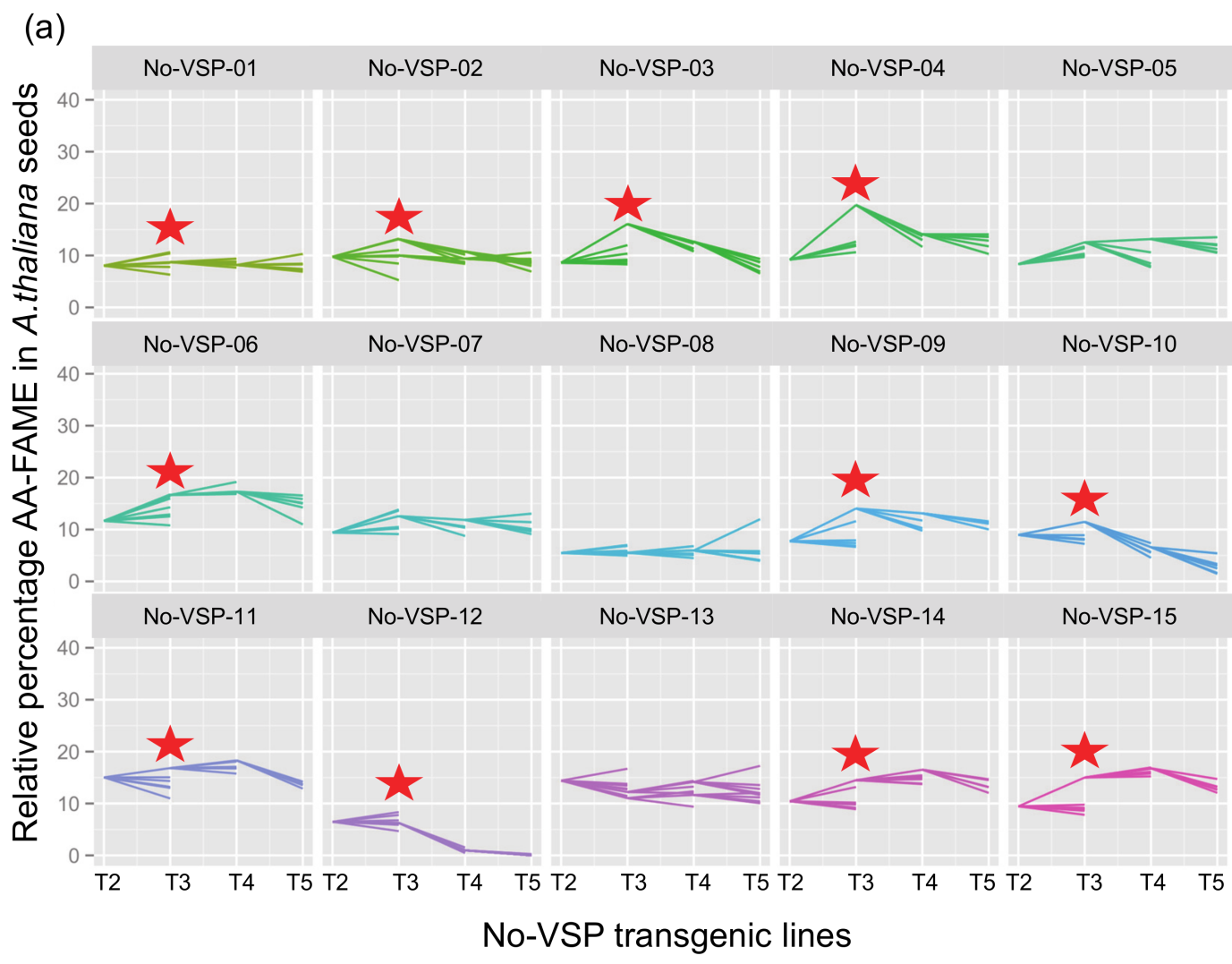


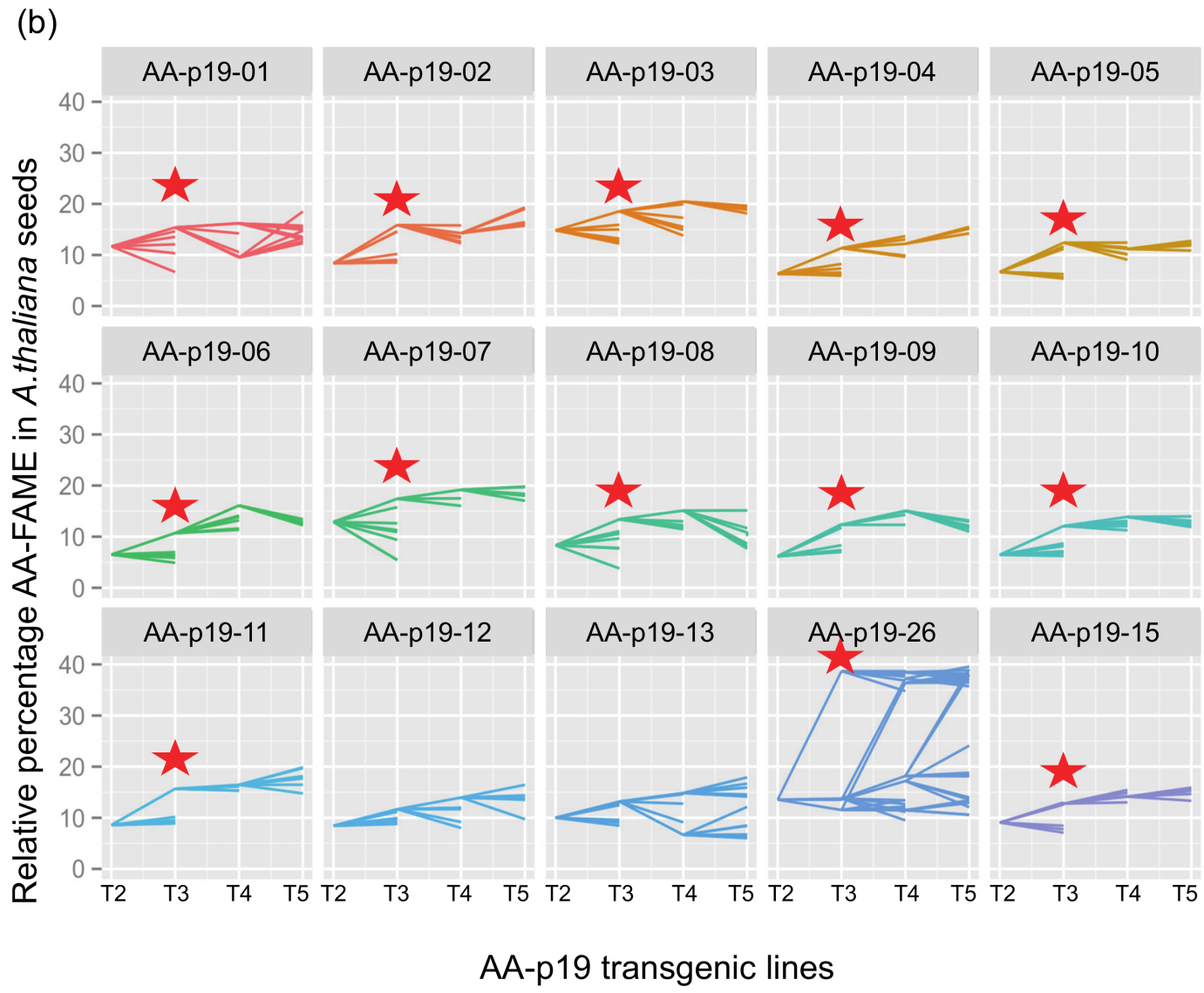


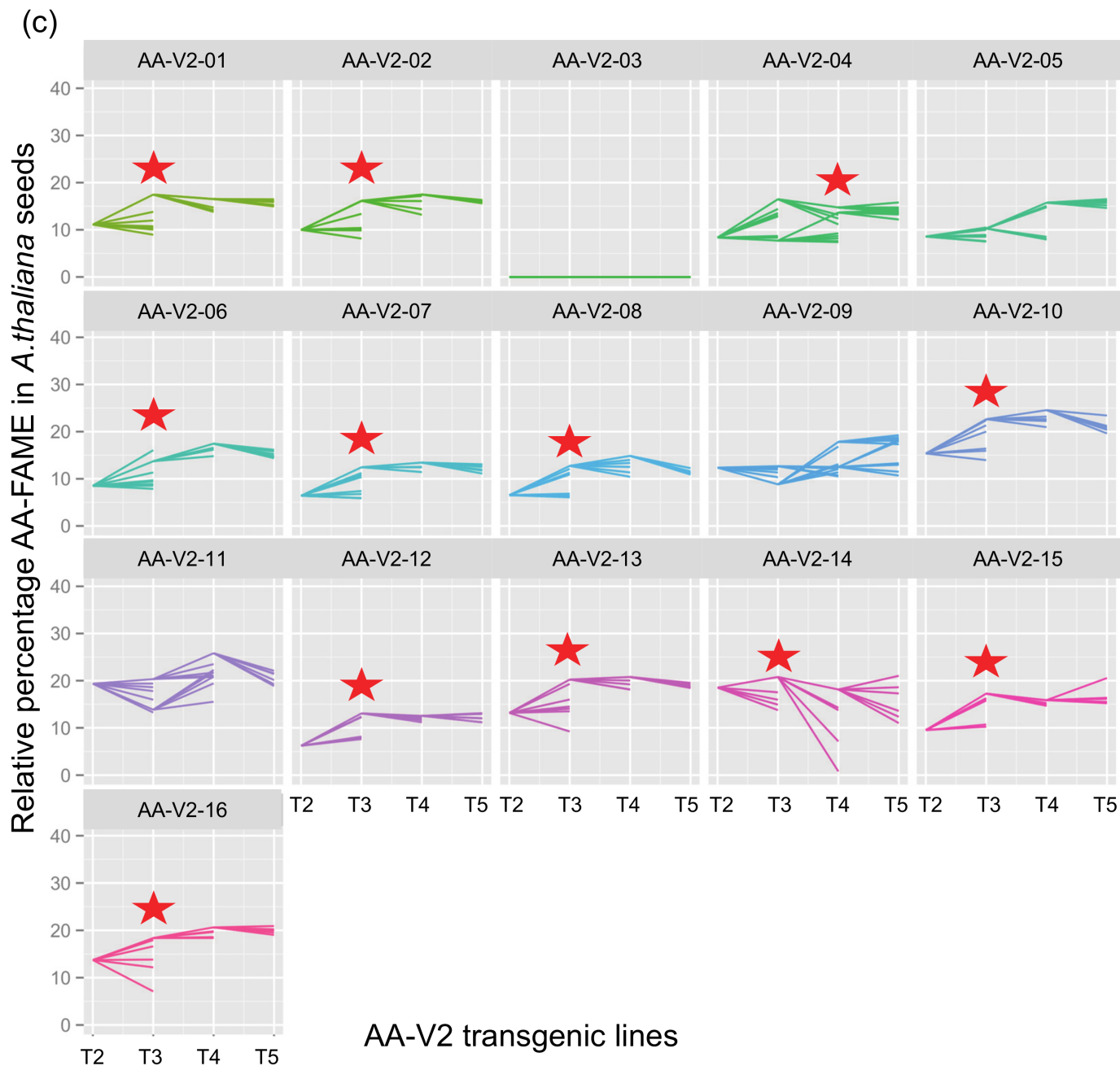


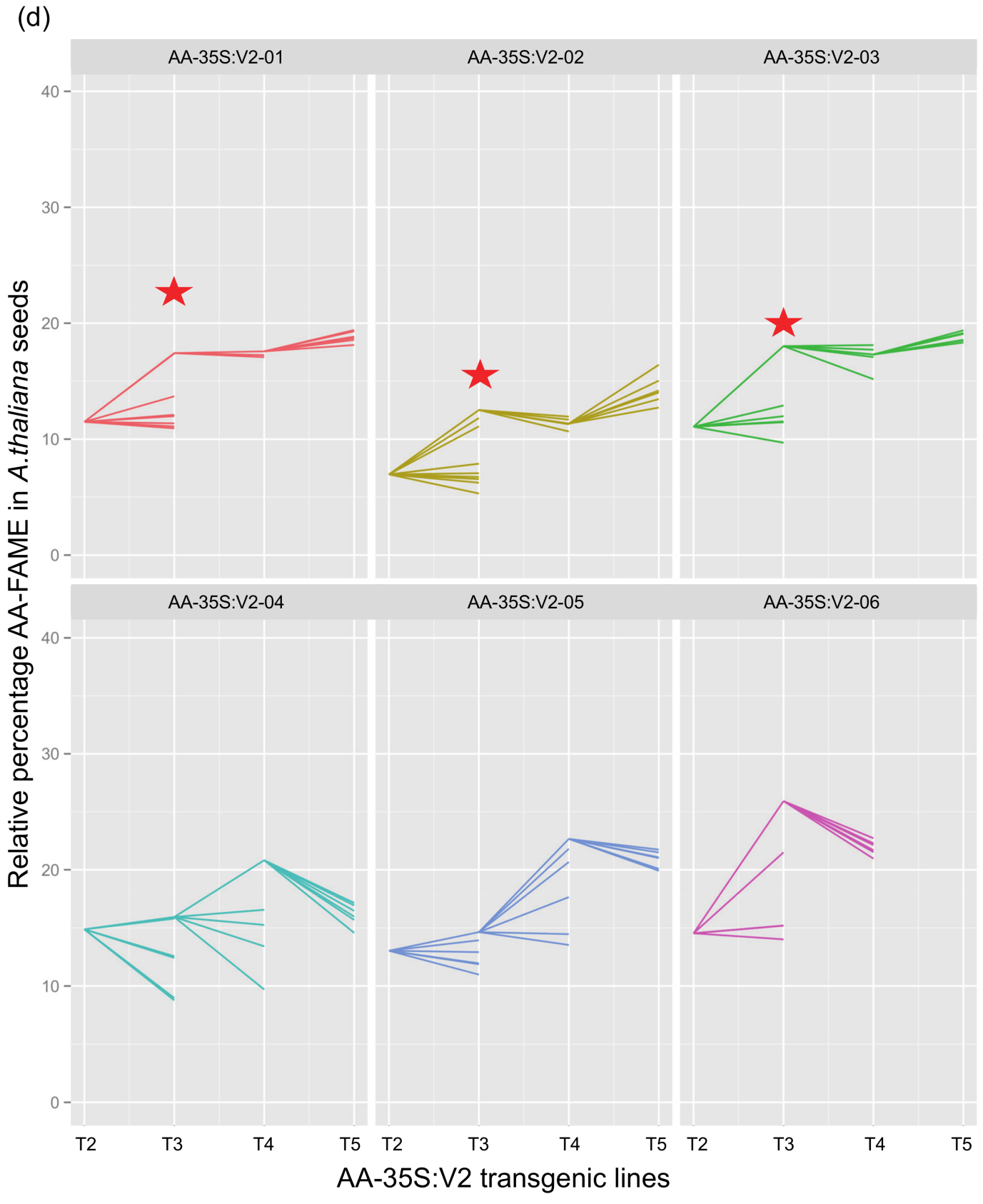

Supplement: Supplementary file 1 — Figure S1 Activity of FP1 promoter measured in different tissues of Arabidopsis thaliana. Figure S2 Dot plot of the percentage of kanamycin resistant T2 seedlings expressing AA. Figure S3 The progeny plots for the various transgenic populations of Arabidopsis thaliana connecting T2 events to their respective progeny in T3, T4 and T5. [file PBI-14-1418-s001.docx]
